# Polish validation of the Transplant Effects Questionnaire

**DOI:** 10.3389/fpsyt.2023.1155672

**Published:** 2023-09-18

**Authors:** Grażyna Dębska, Irena Milaniak, Alicja Dębska-Ślizień, Filip Gołkowski

**Affiliations:** ^1^Faculty of Medicine and Health Sciences, Andrzej FryczModrzewski Krakow University, Krakow, Poland; ^2^Department of Nephrology, Transplantology and Internal Medicine, Gdansk Medical University, Gdańsk, Poland

**Keywords:** emotional response, Transplant Effects Questionnaire, validation, kidney, transplant

## Abstract

**Introduction:**

The Transplant Effects Questionnaire (TxEQ) assesses specific recipients’ reactions to receiving a transplanted organ, including worry about the transplant, Guilt regarding the donor, disclosure of having undergone transplantation, adherence to medical treatment, and responsibility to the donor, family, or medical staff. Poland has no standardized tool for evaluating the emotional reaction to transplantation. The study aimed to assess the basic psychometric properties, such as the reliability and validity of the Polish translation of TxEQ-PL.

**Materials and methods:**

The study involved 84 patients after kidney transplantation. The average age of the subjects was 49.87 years (±15.27). The study used the diagnostic survey method, the Polish versions of the *Revised Life Orientation Test* (LOTR-R), the Mental Component Summary of the SF-36 (SF-36-MCS), and the Hospital Anxiety and Depression Scale (HADS). The Confirmatory Factor Analysis (CFA) was used to verify the factor structure of TxEQ -P.L. variables.

**Results:**

The TxEQ-PL version has satisfactory internal consistency for each subscale (*Cronbach’s alpha* > 0.7). The analysis showed a significant negative relationship between optimism (LOT-R) and the TxEQ-PL subscales: worry about transplant and disclosure of having undergone transplantation and a positive relationship in the subscale of adherence to medical treatment. In addition, a significant negative relationship was found between the subscale: adherence to medical treatment and the severity of depression and intensity of anxiety (HADS); also, a positive relationship with the Mental Component Summary of the SF-36 (SF-36-MCS). The intensity of anxiety and severity of depression were positively related to the TxEQ-PL subscale of disclosure of having undergone transplantation and negatively associated with the Mental Component Summary of the SF-36 (SF-36-MCS). The Confirmatory Factor Analysis confirmed the five-factor structure of the TXEQ-PL questionnaire (RMSEA = 0.083). Not the best fit is indicated by the value of comparative fit indexCFI = 0.813 and SRMR = 0.10. The result of the chi-squared test (220) = 340 was statistically significant; *p* < 0.001.

**Conclusion:**

TxEQ-PL is useful for assessing emotional reactions to organ transplantation. The tool has a factor structure identical to the original English version and comparable psychometric properties.

## 1. Introduction

Organ transplantation is an effective treatment for end-stage renal disease. The success of kidney transplantation in prolonging life and increasing well-being must also be considered in the context of psychosocial aspects of the procedure, as it gives long-term outcomes. The literature analysis shows a need to identify differentiating factors of psychosocial functioning after kidney transplantation (1, 2). Also, little is known about the extent to which transplant recipients struggle with emotional problems. More literature on transplantology emphasizes that receiving an organ may give rise to a new set of stressors, psychological challenges, and adaptive demands. Most often described stressors identified across of transplant population include the side effects of immunosuppressive regiments, fear of organ rejection, worries about the viability of transplanted organs, and the need to adhere to rigorous recommendations after transplantation. Also, other areas influence the well-being of organ recipients. Studies have identified the impact of family relationships, physical and psychosocial adjustment after transplantation, integration of the body image, feelings of gratitude, and Guilt toward the donor or donor’s family (3). The comparison of organ groups allows identification regarding the psychological processing of transplantation experience and adjustment. Studies showed a feeling of responsibility toward family and donors is familiar after transplantation. The heart and lung recipients worry less about their transplant (4–7). However, the correct assessment of the impact of transplantation on the patient’s mental well-being is very important for optimizing treatment (3, 5–7). Poland has no standardized tool for evaluating the emotional reaction to transplantation. The kidney transplant constitutes the largest group of transplant patients in Poland, with a total population of 18,458 recipients. There is growing importance in evaluating the psychological adjustment of this group of transplant recipients to quality of life and depressive and anxiety symptoms. The TxEQ assesses the specific emotional reaction to transplantation. The Transplant Effects Questionnaire (TxEQ) assesses specific recipients’ responses to receiving an organ, including self-care-related issues, i.e., worry about transplant, Guilt regarding the donor, disclosure of having undergone transplantation, adherence to medical treatment and responsibility to the donor, family or medical staff (3, 5–7). The Transplant Effects Questionnaire (TxEQ) can be used to assess these problems quantitatively. The TxEQ was initially developed for kidney transplant recipients and can be used in other organ transplant groups of heart, lung, and liver.

What is more interesting is that the results of TxEQ demonstrated different emotional responses in transplant recipients (3–7). For this purpose, the original English version of TxEQ was translated into Polish. It was verified in a sample of heart transplant recipients concerning the strength of the sense of coherence and coping strategies (3, 8). In the Polish studies, the English-language version of the questionnaire (TxEQ) was translated into Polish by Milaniak et al. (8), taking into account the principle of determining accuracy and equivalence of translation following the guidelines adopted by the World Health Organization (WHO) (9, 10). The Polish version of the questionnaire (TxEQ) adapted by Milaniak et al. required a complete evaluation of the tool’s psychometric properties. The psychometric properties are primarily determined by reliability and validity. Reliability describes the accuracy and extent to which the questionnaire gives consistent and reproducible results. This is determined by evaluating the homogeneity (uniformity) of the measurement tool. The homogeneity of the scale is usually determined by the internal consistency of Cronbach’s *alpha* coefficient (11). Validity answers whether a given tool measures what authors intended it to measure (9, 10). The tools for assessing mental functioning of quality of life with proven psychometric properties are used to evaluate the validity. There is growing evidence that the quality of life of transplant recipients, the symptoms of anxiety and depression, and personal resources like dispositional optimism are closely connected with their ability to psychologically adaption to their new situation (5–7). The relationship between specific emotional reactions measured by the TxEQ was confirmed with quality of life and depressive and anxiety symptoms (5–7). The association between TxEQ and optimism is not fully understood. However, the other personal resources (sense of coherence) were examined (8).

The study aimed to assess the basic psychometric properties, such as the reliability and validity of the Polish translation of TxEQ in the group of kidney transplant recipients and the confirmation of the five-factor structure of the TxEQ-PL. The relationship between the emotional and behavioral response to the received organ and psychological functioning (quality of life, anxiety, depression, and optimism) will be examined to test the construct validity.

## 2. Materials and methods

This work is a part of the multicenter research project titled Nonadherence with therapeutic recommendations among organ recipients—the Occurrence of the Phenomenon and its causes. This substudy was conducted with the University Clinical Centre in Gdańsk from Sep 1, 2018, to Dec 31, 2019. The study was approved by the AFM Krakow University Bioethics Committee (Approval No. KBKA/63/O/2018).

A cross-sectional diagnostic survey and purposive sampling were used. The investigation consisted of a written questionnaire completed by the patients at the outpatient clinic during the routine examination. Patients were enrolled at age ≥ 18, with one organ transplanted, > 1 year after transplant, with good cognitive and medical conditions. The exclusion criteria were: age < 18, more than one transplant, and the patient unable to complete the questionnaire.

### 2.1. Study population

The study involved 84 patients after kidney transplantation. The mean age of the subjects was 49.87 years (*SD* = 15.27). There were 58.8% (*N* = 47) men and 41.2% (*N* = 37) women. Most subjects, 61.2% (*N* = 52), lived in the city. The remaining 39.8% (*N* = 32) lived in rural areas. Almost half of the subjects (49.4%; *N* = 39) were professionally active. The education of the subjects was as follows: 13.1% (*N* = 11)—vocational education, 46.4% (*N* = 39)—secondary education, and 40.5% (*N* = 34)—higher education. The median survival time from kidney transplantation was 8.9 years (SD 6.8) (minimum 1 year, maximum 32 years). The research was carried out in cooperation with the University Clinical Centre in Gdańsk.

To estimate validity, the study used the diagnostic survey method, the Polish versions of the Transplant Effects Questionnaire (TxEQ), *Revised Life Orientation Test* (LOTR-R), Mental Component Summary of the SF-36 (SF-36-MCS), and Hospital Anxiety and Depression Scale (HADS). We included the diverse questionnaires (LOT-R, HADS, SF-36-MCS) because Polish tools with proven psychometric properties are used to estimate theoretical validity.

### 2.2. Transplant Effects Questionnaire

Transplant Effects Questionnaire (TxEQ) is a 23-item scale measuring the emotional reaction important for transplant recipients. The TxEQ consists of five subscales assessing the following: worry about transplant (six items), Guilt regarding the donor (five items), disclosure of having undergone transplantation (three items), adherence to medical treatment (five items), and responsibility to the donor, family or medical staff (four items). The participants are asked to respond to each item on a five-point Likert scale, ranging from “strongly disagree” to “strongly agree” (score from 1 to 5). Subscale scores are expressed as mean obtained by dividing the total score by the number of items, ranging from 1 to 5. Higher scores indicate greater worry about transplant, greater disclosure, more intense feelings of Guilt, greater responsibility, and better adherence to medical treatment. TxEQ norm values are not available. Greater scores show a higher degree for the subscale. Cronbach alpha for the original version of TxEQ was 0.81 for worry, 0.76 for Guilt, 0.86 for disclosure, 0.79 for adherence, and 0.72 for responsibility (3). In this study, we utilized the first Polish version adapted by Milaniak et al. (8). The authors decided to change the negation of sentence question No. 8, “I do not have any feelings of guilt toward the donor,” to an affirmative sentence, “I have any feelings of guilt toward the donor,” and to change the original recode the scoring for answers. According to the scoring algorithms for TxEQ, the answer “strongly agree” is 1, agree is 2, uncertain is 3, disagree is 4, and “strongly disagree” is 5. We recode this item as: “strongly agree” is 5, agree is 4, uncertain is 3, disagree is 2 and “strongly disagree” is 1. In this operation, we do not change the scoring for factor: “Guilt regarding donor” where a higher score indicates more feelings of Guilt.

### 2.3. Revised Life Orientation Test

Revised Life Orientation Test (LOT-R) is an instrument used to assess dispositional optimism, which refers to the stable belief or predisposition of having a generalized view of positive results in the future. The Polish adaptation of the test comprises 10 items with a 5-point Likert scale from 1 (completely disagree) to 5 (completely agree). Three statements were phrased in an optimistic and three in a pessimistic manner. After summing up the scores, the arithmetic mean was calculated, and the result was converted to standardized units on the sten scale and used to evaluate the intensity of dispositional optimism. The results from 1 to 4 sten scores were treated as low and indicated greater pessimism, while those from 7 to 10 sten scores indicated greater optimism. The greater the score obtained in the LOT-R, the higher the degree of dispositional optimism. The reliability of the Polish version of the test is close to the original − 0.87 *Cronbach’s alpha* (12, 13).

### 2.4. Short Form 36 Health Questionnaire

Short Form 36 Health Questionnaire (SF-36) was used for the QOL measurement. It contains 36 items. The SF-36 measures eight scales: physical functioning (10 questions, P.F.), bodily pain (2 questions, B.P.), role physical (4 questions R.P.), general health (5 questions G.H.), vitality (4 questions, V.T.), social functioning (2 questions, S.F.), role emotional (3 questions RE) and mental health (5 questions M.H.). The main concepts measured by the SF-36 are Physical Component Summary (PCS) and Mental Component Summary (MCS). The final measurement included physical activity (physical component summary—PCS) and mental activity (mental component summary—MCS), obtained by adding physical components (P.F., R.P., B.P., VT) for PCS and mental components (S.F., RE, MH, G.H.) for MCS. Scores ranged from 0 to 100. Lower scores show more disability. Higher scores—less disability. *Cronbach’s alpha* coefficients for the Polish version revealed positive internal consistency (0.38–0.98) (14). The Mental Component Summary (MCS) of the SF-36 questionnaire was used for the analysis.

### 2.5. Hospital Anxiety and Depression Scale

Hospital Anxiety and Depression Scale (HADS) by Zigmond and Snaith (15), adapted by Majkowicz (16), was used to assess the intensity of anxiety and severity of depression. The HADS scale consists of two independent subscales measuring the level of anxiety and severity of depression. Each subscale contains 7 statements regarding the subject’s current state, which can be rated from 0 to 3 points. Test reliability of the HAD-Scale for own research measured by the coefficient Alfa Cronbacha was–for anxiety 0.76 and depression −0.74.

Statistical analyses were carried out using the IBM SPSS Statistics 27 package and Jamovi software to answer the research questions. The tools were used to analyze basic descriptive statistics, conduct *r*Pearson correlation analysis, and confirmatory factor analysis (CFA). In the model fit evaluation using confirmatory factor analysis, the following measures of fit were considered: RMSEA, SRMR, and CFI. The value of RMSEA, SRMR below 0.05 indicates a good fit with the data, below 0.08—a satisfactory fit, and above 0.10—a poor fit of the model. The CFI can be rated from 0 to 1, where 1 is the best fit, and > 0.9 is a good fit with the data (17, 18).

The level of significance was *α* = 0.05. The result of the Shapiro–Wilk test was statistically significant for most variables, which means that their distributions significantly differ from the normal distribution. Therefore, it is reasonable to conduct an analysis based on parametric tests.

## 3. Results

### 3.1. Psychometric analysis of the Polish version of the TxEQ-PL questionnaire

No missing values or outliers were found at the descriptive level of the TxEQ. The mean for 23 items ranged from 1.0 ± 0.65 to 5 ± 0.73, skewness from −0.74 to 0.05, and kurtosis from −0.69 to −0.23. For most variables, the skewness of the distribution does not exceed the conventional absolute value of 1; therefore, the distribution of variables should be treated as normal (Table 1).

**Table 1:** Basic descriptive statistics of the variables (*N* = 84).

	*M*	*Me*	*SD*	*Sk*	*Kurt*	*Min*	*Max*
Mental component summary (SF-36-MCS)	47.21	47.22	7.81	−0.27	−0.29	25.26	60.84
Optimism (LOT-R)	16.11	16.00	4.02	−0.15	−0.35	6.00	24.00
Anxiety (HADS-A)	4.84	5.00	3.29	0.34	−0.73	0.00	12.00
Depression (HADS_D)	3.27	3.00	2.97	0.90	0.18	0.00	12.00
Worry (TxEQ)	3.23	3.33	0.76	0.05	−0.31	1.50	5.00
Guilt (TxEQ)	2.01	2.00	0.65	0.25	−0.37	1.00	3.80
Disclosure (TxEQ)	1.90	1.67	0.78	0.61	−0.69	1.00	3.67
Adherence (TxEQ)	4.25	4.50	0.73	−0.74	−0.48	2.40	5.00
Responsibility (TxEQ)	3.41	3.50	0.92	−0.43	−0.23	1.00	5.00

### 3.2. The assessment of the reliability of the Polish version of the TxEQ-PL questionnaire

The TxEQ-PL version showed satisfactory internal consistency (Cronbach’s alpha: worry about transplant *α* = 0.69, Guilt *α* = 0.71, disclosure *α* = 0.76, adherence *α* = 0.76, responsibility *α* = 0.78) (Table 2). The reliability of the TxEQ-PL is confirmed by the satisfactory values of the Intraclass Correlation Coefficient (ICC) obtained for the subscales. ICC was for worry 0.695; 95% CI [0.578–0.788]; for guilt 0.711; 95% CI [0.597–0.800]; for disclosure 0.757; 95% CI [0.648–0.837]; for adherence 0.764; 95% CI [0.671–0.837]; and for responsibility 0.787 95% CI [0.699–0.854] (19).

**Table 2:** Cronbach’s alpha for the TxEQ- Polish and the English version.

	English (3)	Polish (4)	Polish-PL
Worry	0.81	0.61	0.69
Guilt	0.76	0.63	0.71
Disclosure	0.86	0.72	0.76
Adherence	0.79	0.61	0.76
Responsibility	0.72	0.63	0.78

However, when conducting the current pilot study, the questions from the Polish version were analyzed in terms of understanding and interpretation following the key. The authors decided to change the negation of sentence question No. 8, “I do not have any feelings of guilt toward the donor,” to the affirmative sentence, “I have any feelings of guilt toward the donor,” and to change the original recode the scoring for answers. According to the scoring algorithms for TxEQ, the answer “strongly agree” is 1, agree is 2, uncertain is 3, disagree is 4, and “strongly disagree” is 5. We recode this item as: “strongly agree” is 5, agree is 4, uncertain is 3, disagree is 2, and “strongly disagree” is 1. In this operation, we do not change the scoring for factor: Guilt regarding donor where a higher score indicates more feelings of Guilt. The recoded question No. 8 was more understandable for the authors and positively impacted the reliability coefficient for the “worry” subscale.

### 3.3. The evaluation of the validity of the Polish version of the TXEQ-PL questionnaire

The relationship was assessed between individual factors/subscales of the TxEQ-PL questionnaire, optimism, intensity of anxiety and severity of depression, and the mental domain of quality of life—Mental Component Summary (MCS) of the SF-36. *r*Pearson correlation was performed to check the relationships of the TxEQ-PL subscales: worry, Guilt, disclosure, adherence, and responsibility with optimism (LOT-R), quality of life (SF-36-MCS), the intensity of anxiety and severity of depression (HADS). The analysis showed a statistically significant negative relationship between worry and optimism. Moreover, in the TxEQ-PL, disclosure was significantly, negatively, and moderately strongly associated with optimism and mental quality of life and positively connected with the severity of depression and intensity of anxiety. Adherence was significantly, positively, and weakly related to mental quality of life and optimism, while negatively and weakly associated with the severity of depression and intensity of anxiety (Table 3).

**Table 3:** The relationship between the factors/subscales of the TxEQ-PL questionnaire, optimism, intensity of anxiety and severity of depression, and the mental domain of the quality of life (*N* = 84).

		Worry	Guilt	Disclosure	Adherence	Responsibility
(LOT-R)	*r*Pearson	−0.25	−0.14	−0.42	0.29	−0.09
	Correlation coefficient	**0.027**	0.243	**<0.001**	**0.011**	0.468
(HADS-A)	*r*Pearson	0.20	0.06	0.26	−0.26	−0.04
	Correlation coefficient	0.076	0.603	**0.024**	**0.020**	0.762
(HADS-D)	*r*Pearson	0.14	0.12	0.31	−0.24	0.00
	Correlation coefficient	0.225	0.280	**0.006**	**0.039**	0.994
(SF-36-MCS)	*r*Pearson	−0.14	−0.12	−0.38	0.24	0.00
	Correlation coefficient	0.238	0.319	**<0.001**	**0.031**	0.994

Bold values means *p < 0.05.

### 3.4. Confirmatory factor analysis of the TxEQ-PL questionnaire

To confirm the five-factor structure of the TxEQ-PL questionnaire, Confirmatory Factor Analysis (CFA) was performed using the JAMOVI software.

In general, the comparative fit indices’ values confirm the tool’s five-factor structure. The value of RMSEA (root mean square error of approximation) was 0.083, which can be considered an acceptable value, as it slightly exceeded 0.08.

**Figure 1:**
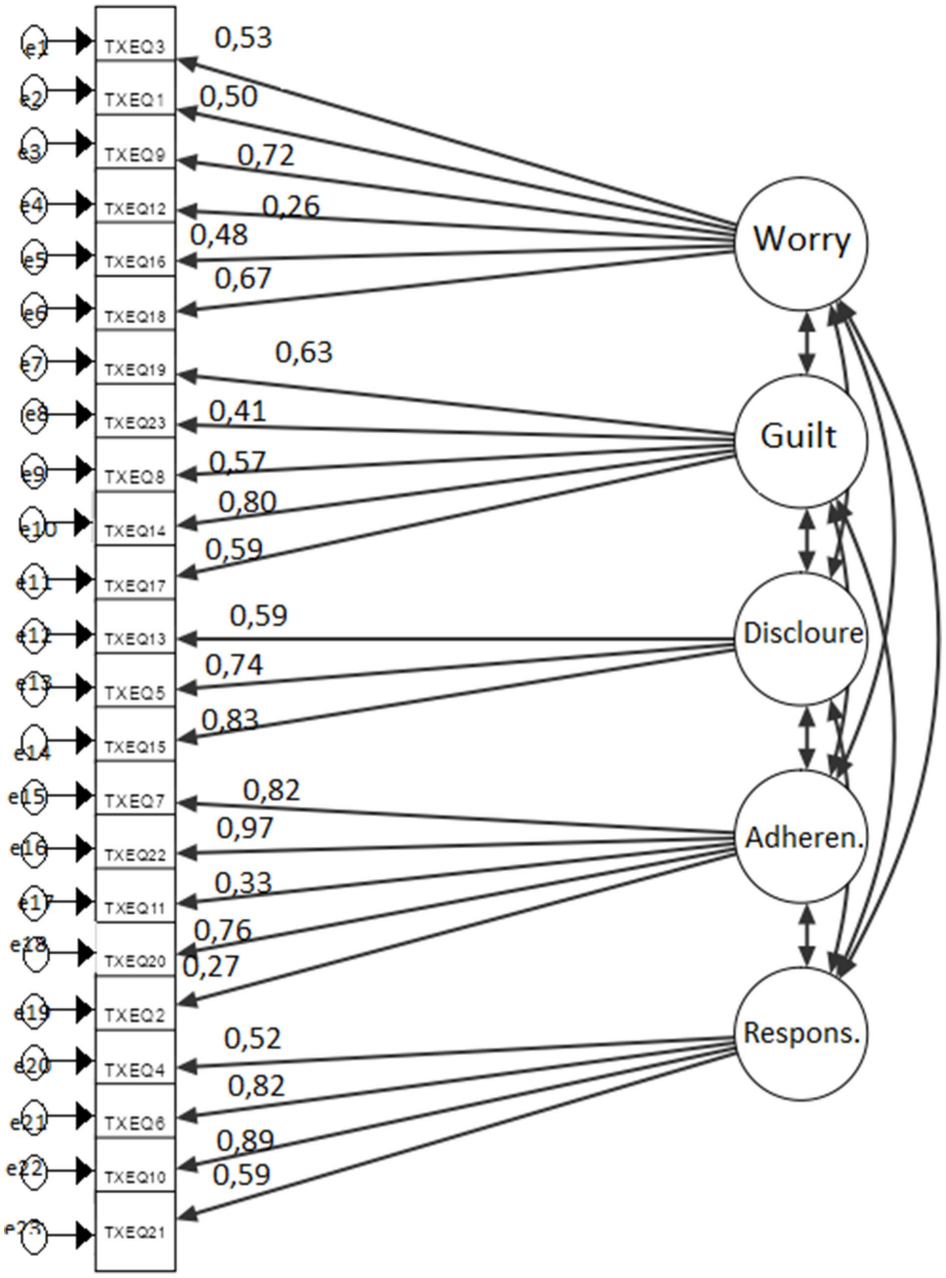
The results of confirmatory factor analysis for the TxEQ-PL questionnaire. Three TxEQ-PL items showed factor loadings <0.40.

Not the best fit is indicated by the value of comparative fit index CFI = 0.813 and SRMR = 0.10, which is the limit value, above which the result indicates a poor fit of the model. The chi-squared test (220) = 340 was statistically significant; *p* < 0.001, indicating a discrepancy between the covariance matrix observed in the study and that implied by the model. The model and standardized regression coefficients are shown in Figure 1. Standardized coefficients for the relationships between the factors are presented in Table 4 for better clarity.

**Table 4:** The relationships between the 5 factors of the TxEQ-PL scale—Polish version.

		Worry	Guilt	Disclosure	Adherence	Responsibility
Worry	Stand. estim.	—				
Guilt	Stand. estim.	0.48	—			
Disclosure	Stand. estim.	0.37	0.49	—		
Adherence	Stand. estim.	−0.11	−0.23	−0.41	—	
Responsibility	Stand. estim.	0.71	0.53	0.18	−0.20	—

The tested model was re-evaluated by applying CFA (Confirmatory Factor Analysis), excluding three items with a factor below 0.4. The obtained indicators of goodness of fit were better and confirmed the five-factor structure of the tool—the structure of the TxEQ. And so, the value of the RMSEA indicator = 0.075; CFI = CFI 0.879; SRMR 0.077. The Chi-square test (160) = 231 result was also significant; *p* < 0.001. These were the following items: 12—“I monitor my body more closely than before I had the transplant-subscale “worry,” 2—“Sometimes I think I do not need my anti-rejection medicines: and 11—“I find it difficult to adjust to taking my prescribed anti-rejection drug regime—“adherences” subscale.

## 4. Discussion

Transplantation has a significant impact on the physical and mental well-being of patients. Standardized tools, such as the Transplant Effects Questionnaire (TxEQ), are used to assess and compare these effects and help optimize treatment. TxEQ adds a new dimension to the measurement of the psychological functioning of transplant recipients. The TxEQ tool evaluates five conceptually coherent factors: worry about transplant, Guilt regarding the donor, disclosure of having undergone transplantation, adherence to medical treatment, and responsibility to the donor, family, or medical staff. The research results show that organ transplant recipients experience various emotions; for instance, they achieve a high degree of adherence to medical treatment and responsibility. They have no problem disclosing the fact of having undergone transplantation; they worry about the transplanted organ and do not feel guilty toward the donor or their family (3–8, 20, 21).

The original English version of the questionnaire was developed and tested among kidney transplant recipients (3). Later, it was translated into other languages. At present, there are various language versions: German, Spanish, and Dutch, for recipients of kidney transplants from a living or deceased donor and recipients of the heart and liver (7, 8, 20, 21).

The German version (TxEQ-D) was validated in the group of heart, lung, liver, and kidney transplant recipients (20); the Dutch version (TxEQ-NL)— among liver transplant recipients (21), the Polish version—in the group of heart transplant recipients (8) and the Spanish version—in a sample of 240 liver transplant recipients (7). This study used the Polish language version of the Transplant Effects Questionnaire (TxEQ) developed by Milaniak et al. (8) and based on the original English version (3). In the study conducted by Milaniak et al. (8). *Cronbach’s alpha* was low in the “worry” subscale (0.63) and in the current study − 0.71, which guaranteed meeting the criterion of reliability. A satisfactory value of *Cronbach’s alpha* is considered at least 0.70 (6, 22). The literature shows that a satisfactory *Cronbach’s alpha* coefficient, i.e., between 0.71 and 0.79, was reported in the German (19), Dutch (0 0.66–0.79) (21), Polish (0.61–0.72) (8) and Spanish version (0.77–0.91) compared to the original English version (0.72–0.86) (3, 7).

The factor structure of the versions is similar to the original English version, consisting of five subscales: worry, Guilt, disclosure, adherence, and responsibility. Using a scree graph, the German version confirmed the five-factor structure in Confirmatory Factor Analysis (CFA) (20). In the Dutch and Spanish versions, RMSEA = 0.063 showed a good fit of the model (7, 21). In their work on the Polish version, Milaniak et al. did not present comparative fit indices (8). However, in own research into the Polish version, the TxEQ-PL Confirmatory Factor Analysis revealed a five-factor structure corresponding to the original English version (3). The three items (2,11,12) of the TxEQ-PL showed factor loadings <0.40, and the Dutch version with for items (2,8,12,19)- had factor loadings <0.40 (21).

In own research, theoretical validity was estimated using correlation analysis and confirmatory factor analysis. Weak correlations were demonstrated between the Polish version of the TXEQ and the general measures of psychosocial functioning, such as optimism, quality of life regarding mental functioning, intensity of anxiety, and severity of depression. The results indicated that the related to the severity of depression and intensity of anxiety (5, 23–27).

TxEQ is an important and reliable research tool for measuring the emotional reaction of organ transplant recipients. It enables the identification of transplant recipients with emotional problems caused by receiving an organ and offering them appropriate support. Own research is important in assessing the emotional reactions to transplantation in clinical practice. Citing the work of Serafini et al. (28) can be assumed that patients who presented with depression may even be at risk for treatment resistance and, thus, suicidal behavior. Notably, compared to non-suicidal subjects, higher mean concentrations of inflammatory mediators have been found in both the periphery and brain of individuals at risk for suicide (28). When analyzing the literature, attention should be paid to the work of Berardelli et al. (29), which showed a relationship between the transplant and adverse effects such as suicidal behavior. In particular, it has been reported that the hypothalamic–pituitary–adrenal (HPA) axis activity is involved in suicide risk, regardless of the presence or absence of psychiatric conditions, even in those who need to carry out kidney transplantation. Moreover, the HPA axis abnormalities, mainly characterized by hyperactivity of the HPA axis, may exert an important modulatory influence on suicide risk, and impaired stress response mechanisms contribute to suicide risk. Therefore, TxEQ-PL can be used to assess emotional reactions to transplantation to assess the risk of suicidal behavior, which can be used in psychotherapeutic practice.

Studies aimed at validating TxEQ in a group of other organ transplant recipients are also justified to test sensitivity and specificity and assess changes over time. Using TxEQ-PL as a screening tool should be done with caution. This is because the size of the group is important to evaluate the tool’s reliability (a group of more than 300 subjects is recommended) (30). In the Polish studies, the group size was smaller than recommended. In the current study − 84 subjects, in the study conducted by Milaniak *N* = 46 (8). The groups in the studies validating the Spanish version were more numerous – 240 and 281 in the Dutch version (7, 21). Over 300 subjects participated in the validation of the TxEQ in England (*N* = 336) and Germany (*N* = 370) (3, 20). However, the results of our research can be considered credible, considering that the views on the required sample size for CFA are different; it can be assumed after Barrett and Kline (31) that the sample size of 50 people is sufficient.

### 4.1. Limitations of the study

Our study has some limitations. The first sample consisted of kidney transplant recipients from one transplant center; the adaptation and validation cannot be transferred to other organs like the lungs or liver. Second, all kidney transplants were from deceased donors, and the results could differ in living donor kidney transplants.

## 5. Conclusion

TxEQ-PL is a valuable tool to assess problems related to emotional reactions to organ transplantation. It can be used in psychotherapeutic practice in evaluating kidney transplant recipients. TxEQ-PL is an essential measure of the psychological functioning of transplant recipients. TxEQ-PL—the Polish version has a factor structure identical to the original English version and comparable psychometric properties. The TxEQ allows for future investigation of the psychological adjustment to transplantation to other important variables like quality of life, depressive symptoms, and adherence.

## Data availability statement

The raw data supporting the conclusions of this article will be made available by the authors, without undue reservation.

## Author contributions

GD and IM: conception and design of the research, collection of data, data analysis, and interpretation. AD-Ś and FG: drafting the manuscript and making intellectual contributions on text and revisions, final approval of the manuscript. All authors contributed to the article and approved the submitted version.

## Funding

The research was co-financed from funds allocated for scientific activity of the Andrzej Frycz Modrzewski Krakow University and the fund of the Medical University of Gdańsk.

## Conflict of interest

The authors declare that the research was conducted in the absence of any commercial or financial relationships that could be construed as a potential conflict of interest.

## Publisher’s note

All claims expressed in this article are solely those of the authors and do not necessarily represent those of their affiliated organizations, or those of the publisher, the editors and the reviewers. Any product that may be evaluated in this article, or claim that may be made by its manufacturer, is not guaranteed or endorsed by the publisher.
